# Mechanical Circulatory Support as a Bridge to Lung Transplantation: A Single Canadian Institution Review

**DOI:** 10.1155/2017/5947978

**Published:** 2017-08-29

**Authors:** Katie Kinaschuk, Sabin J. Bozso, Kieran Halloran, Ali Kapasi, Kathy Jackson, Jayan Nagendran

**Affiliations:** ^1^Faculty of Medicine and Dentistry, University of Alberta, Edmonton, AB, Canada; ^2^Department of Surgery, Division of Cardiac Surgery, Edmonton, AB, Canada; ^3^Department of Medicine, University of Alberta, Edmonton, AB, Canada; ^4^Human Organ Procurement and Exchange Program, Edmonton, AB, Canada; ^5^Canadian National Transplant Research Program, Edmonton, AB, Canada; ^6^Alberta Transplant Institute, Edmonton, AB, Canada

## Abstract

**Background:**

Lung transplant (LTx) waitlists continue to grow internationally. Consequently, more patients are progressing to require mechanical circulatory support (MCS) as a bridge to transplantation (BTT). MCS strategies include interventional lung assist (iLA) and venovenous (VV) and venoarterial (VA) extracorporeal membrane oxygenation (ECMO). We review our series of patients bridged with MCS while listed for LTx.

**Methods:**

All consecutive patients, listed for LTx requiring MCS as a BTT at the University of Alberta from 2004 to 2015, were included. Patient demographics and outcomes were compared for the 3 groups (iLA, VV-ECMO, and VA-ECMO).

**Results:**

Of the 24 patients supported with MCS devices, 17 were successfully transplanted and 7 died waiting. In total, 25% (*n* = 6) were bridged with VA-ECMO, 54% (*n* = 13) with VV-ECMO, and 21% (*n* = 5) with iLA. Overall, 71% of patients were bridged successfully to LTx. The 1-year survival posttransplantation was 88%.

**Conclusion:**

We have demonstrated the feasibility of utilizing the MCS modalities of VA-ECMO, VV-ECMO, and most recently iLA, as a BTT. MCS is a viable strategy for BTT, offering improved survival outcomes for decompensating adult patients awaiting LTx, resulting in excellent survival posttransplantation.

## 1. Introduction

Over the past several decades, lung transplantation (LTx) has increased significantly as a treatment for end-stage lung disease [1]. As reported by the International Lung Transplant Registry, from 2000 to 2015, a 2.5-fold increase was seen in the number of lung transplants performed internationally. Though incidence of lung transplantation is increasing rapidly, the number of available donor organs has remained an obstacle, with donor organs remaining at a number inconsistent with the growing need. This discrepancy has resulted in long recipient waitlist times, contributing to increased recipient morbidity, and increased waitlist mortality [2]. Presently, waitlist mortality at major North American transplant centres remains at approximately 15–30% [3].

The University of Alberta is the largest tertiary care transplant centre in Western Canada, servicing a catchment size of over 6-million km and approximately 7-million people. Over the past decade, the University of Alberta has seen a significant increase in the number of lung transplantations performed, from 42 in 2010 to 61 in 2015. The average waitlist time at our centre has decreased over the last two decades, with times now comparable with other North American centres at 200 days and an average waitlist mortality of 32%. Survival has been respectable in our experience, with 88% 1-year survival overall posttransplantation.

Mechanical circulatory support (MCS) devices were first introduced in 1950 for cardiac failure, later to be used in pulmonary failure. MCS strategies previously regarded as rescue therapies are more commonly being employed in transplant medicine, specifically in cardiothoracic transplantation as a bridge to transplant (BTT) [4, 5].

It is now recognized that the use of MCS devices can produce satisfactory 1-year survival outcomes for patients requiring MCS bridging prior to their LTx [6, 7]. MCS devices used clinically in transplantation currently include ventricular assist devices, venoarterial and venovenous extracorporeal membrane oxygenation (ECMO), and interventional lung assist (iLA). The iLA is a pumpless low-resistance device that is connected arteriovenously and has been used as a BTT primarily in patients with ventilation-refractory hypercapnic lung failure. This device very effectively removes carbon dioxide while improving oxygenation only marginally. Several studies have demonstrated the feasibility of iLA as a BTT with a variety of cannulation strategies being employed, depending on the etiology of the respiratory failure [8, 9].

Herein, we report our single-centre experience with bridging patients using MCS while listed for LTx.

## 2. Methods

### 2.1. Study Design

From January 1, 2004, to December 31, 2015, twenty-four consecutive patients were bridged to double-lung transplantation using MCS with either VV-ECMO, VA-ECMO, or iLA at the UAMHI. The present study involved a single-institution retrospective cohort with the intention of evaluating the overall outcomes of patients bridged to transplant with MCS.

### 2.2. Donors and Recipients

The lungs used in these cases were all donated after neurological declaration of death. They were provided through the Human Organ Procurement and Exchange (HOPE) program. All recipients were deemed eligible for LTx due to end-stage pulmonary disease. All recipients gave informed consent and the study protocol was approved by the local Research Ethics Board. Recipient charts were evaluated for baseline clinical characteristics and followed for survival.

### 2.3. MCS Protocol

Allocation of MCS strategy was based on institutional evidence and best practice. The decision to institute MCS was based on institutional knowledge in patients with end-stage respiratory failure refractory to maximal medical therapy. Modality selection was based on a multidisciplinary team conference involving transplant respirologist, transplant surgeons, critical care extracorporeal life support (ECLS) team, social work team, dietary team, and the infectious disease team.

## 3. Results

### 3.1. Baseline Characteristics

The study sample included 24 consecutive patients undergoing MCS bridging while awaiting double LTx from January 1, 2004, to December 31, 2015. Baseline demographic data is summarized in Table 1, stratified by MCS modality.

Of the patients bridged with MCS, the distribution of modality of MCS was VV-ECMO 54% (*n* = 13), VA-ECMO 25% (*n* = 6), and ILA 21% (*n* = 5). Based on the small sample size, there were not any significant differences in age or gender, though females were more likely to be bridged compared to patients undergoing transplantation over the same time-period (*p* < 0.05). Further demographics, including diagnosis and rates of successful bridge to transplant, are described in Table 1.

### 3.2. Outcomes

**Figure 1:**
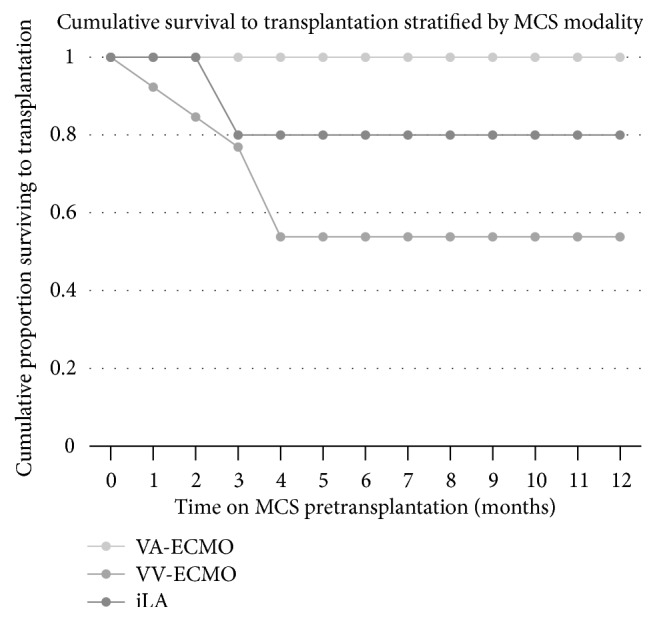


**Figure 2:**
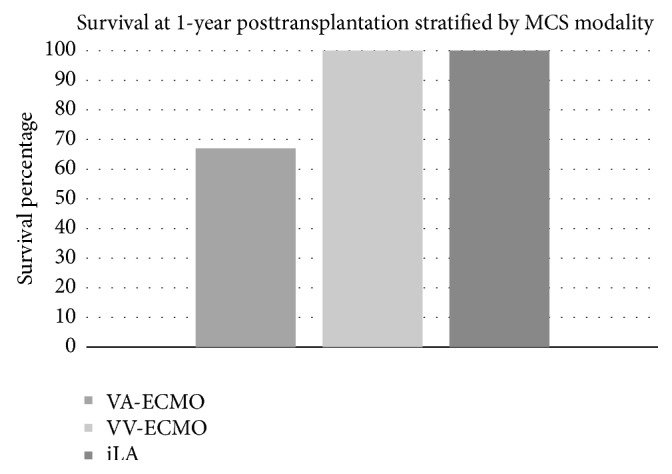


Outcomes for patients who had MCS bridging instituted are summarized in Table 2. Patients bridged with iLA had the longest mean waitlist time (830 days) and the longest mean bridge time (45 days), compared to VV-ECMO and VA-ECMO (*p* < 0.05). Donor age was greatest in the VA-ECMO recipients (43.2-years) compared to VV-ECMO (29.1-years) and iLA (36.8-years). Actuarial survival to transplantation is shown in Figure 1. Overall, 53% of VV-ECMO, 100% of VA-ECMO, and 80% of iLA patients survived to transplantation. Posttransplantation ICU length of stay did not differ significantly between groups (*p* > 0.05). Primary graft dysfunction (PGD) Grade 3 occurred in 29% (*n* = 2) of VV-ECMO recipients, 25% of VA-ECMO recipients, and none of the iLA recipients at 72-hour posttransplantation. Survival at 1-year posttransplantation is shown in Figure 2 and was 100% in both the VV-ECMO and iLA groups and 67% in the VA-ECMO group. Overall survival at 1-year posttransplantation was 88%. Causes of death are described in Table 2.

## 4. Discussion

As there is a growing population of potential recipients that may benefit from lung transplantation, there is an increasing need for bridging strategies in patients that develop end-stage respiratory failure while awaiting lung transplantation. The use of mechanical circulatory support (MCS) bridging remains controversial with limited reported outcomes in the literature. Our experience has also been limited, though similar in size to current reported results [6, 7]. Mason et al. described that there was decreased survival in patients undergoing MCS bridging that is attributed to the perioperative phase; however, patients surviving past 6 months have comparable survival to those patients not requiring pretransplantation MCS [6]. It is important to recognize that this finding may likely be attributed to the acute nature and care of patients requiring BTT, as opposed to the use of MCS devices themselves [10].

The outcomes from the use of MCS as a BTT have improved over time from prior reports. Our results lend support to the feasibility of MCS bridging with an excellent overall 1-year survival of 88% in those patients requiring MCS bridging. This study provides a contemporary analysis of data at a centre with lung transplant volumes within the top 10% of the ISHLT registry database [11] and significant experience utilizing the MCS modalities described. We believe that the improvement in outcomes after MCS bridging is likely due to increased experience and confidence with this approach, advancements in both medical and surgical care, and the development of an experienced multidisciplinary team including transplant respirologists, transplant surgeons, critical care teams, social workers, dieticians, and infectious disease specialists. Furthermore, in our series of bridging patients, rates of PGD at 72 hours (12.5%) were very acceptable in our cohort.

The consideration for the use of MCS strategies as BTT has evolved over time. In recent guidelines, initiation of MCS in hemodynamically marginal patients is recommended to prevent posttransplant outcomes that may be adversely affected by the development of irreversible end-organ dysfunction [12]. Patient selection is determined based on a multidisciplinary discussion as aforementioned. The criteria for MCS modality are also reached by multidisciplinary consensus. VA and VV-ECMO are dependent on cardiac function with VA-ECMO being indicated in combined cardiac and pulmonary failure and VV-ECMO being indicated in isolated respiratory failure. iLA bridging is primarily indicated in patients with ventilation-refractory hypercapnic lung failure. However, due to the pumpless nature of this device, sufficient system perfusion pressure must be maintained. Thus, indications for iLA institution have traditionally been in patients with respiratory failure secondary to pulmonary hypertension. There is potential for extended use beyond these indications, including patients with secondary pulmonary hypertension from other pulmonary diseases including idiopathic pulmonary fibrosis and cystic fibrosis. The outcomes of this study suggest that, in appropriately screened potential recipients, initiation of MCS can lead to acceptable rates of both bridging to transplantation and survival posttransplantation.

There were differences in rates of successful BTT based on type of MCS modality. VV-ECMO portended the worst likelihood of a successful bridge (*n* = 7; 53%) despite providing 100% posttransplantation 1-year survival. By comparison, all patients bridged with VA-ECMO underwent LTx; however, only 67% of recipients were alive at 1 year. This finding likely reflects the acute, poor functional status of those patients requiring VA-ECMO support, leading to variability in organ allocation. The poor survival posttransplantation of those bridged with VA-ECMO highlights the importance of ensuring appropriate organ allocation to recipients with the greatest chance of a good outcome. This may require further reevaluation of suitability for transplantation with weekly discussions of the multidisciplinary team. This approach has now been developed at our program for patients bridged on MCS.

Patients undergoing BTT with the iLA device endured the longest amount of time on the waitlist (mean 830 days) and underwent the longest bridge time (mean 44.8 days). This is likely due to the prevalence of PPH in these patients and difficulty in finding suitable donor organs, especially when combined heart-lung transplantation is indicated. In fact, our centre has recently reported the longest such successful bridge using iLA in a PPH recipient undergoing combined heart-lung transplantation [13]. We anticipate future increased utilization and success for BTT with iLA resulting from prolonged bridge support capability and notable transplant survival outcomes, with 100% of those transplanted following iLA bridging at our institution being alive to date.

The previously described pumpless iLA has the potential for expanded use, potentially in patients with secondary pulmonary hypertension, as well as its indicated use in patients with specified primary pulmonary hypertension. We believe that the shift towards iLA use over ECMO may improve pre-, peri-, and posttransplant outcomes, as the pumpless iLA theoretically eliminates some of the consequences associated with pump-dependent ECMO systems.

This study is not with limitations. Weaknesses include the modest sample population size of 24. These moderate numbers can be attributed to the select population of patients awaiting transplant who are eligible based on device inclusion criteria, to be initiated on one of the previously described MCS modalities. As well, our results represent the retrospective Western-Canadian experience of the past decade. However, with improved technologies, the potential for expansion of indications for different modalities of MCS may improve outcomes with further experience and refinement within the field. We expect this to be particularly true for the iLA device, which offers a pumpless system, theoretically ameliorating some of the drawbacks of the pump-dependent ECMO systems.

## 5. Conclusion

In the University of Alberta experience, MCS as a BTT offers a viable strategy for improving survival outcomes of decompensating adult patients awaiting lung transplantation. The use of MCS technologies at this institute over the past decade has led to a reduction in waitlist mortality, with very good 1-year posttransplant survival rate of 88%, for those who underwent pretransplant MCS. We believe the utilization of MCS bridging devices, based on our institutional evidence, to be a viable strategy to decrease waitlist mortality in high acuity patients awaiting LTx.

## Figures and Tables

**Table 1:** Patient characteristics.

Characteristics	VV-ECMO	VA-ECMO	iLA
	(*n* = 13)	(*n* = 6)	(*n* = 5)
Age in years	44.3	41.1	49.5
Female (%)	46	67	60
BMI	25.7	22.6	22.9
Smoking history (%)	54	33	20
Successfully bridged (%)	53	100	80
Diagnosis			
IPF	5	1	0
PPH	0	1	3
ILD	2	1	1
CF	2	2	0
Sarcoidosis	1	0	0
Others	3	1	1

All numbers provided are means unless otherwise stated.

**Table 2:** Peri- and posttransplantation characteristics.

Characteristics	VV-ECMO	VA-ECMO	iLA
	(*n* = 7)	(*n* = 6)	(*n* = 4)
Waiting list time (d)	95	36	830
Donor age (y)	29.1	43.2	36.8
Cold ischemic time			
Right lung (min)	321	406	262
Left lung (min)	342	454	305
ICU LOS (d)	13.1	14.7	16.5
Hospital LOS (d)	49.7	41	61.5
CPB use (%)	78	100	100
Intubation (d)	12.9	14.7	10.2
PGD 3 at T72	2 (29%)	1 (25%)^*∗*^	0 (0%)
Bridge time (d)	17.9	19.2	44.8
Survival at 1 year (%)	100	67	100
Overall causes of death			
Sepsis	1	—	—
BOS	1	—	—
Gastrointestinal	—	1	—
Others	—	1	—

^*∗*^PGD 3 missing in 2 VA-ECMO. All numbers provided are means unless otherwise stated.
